# Evaluating the Accuracy of Low-Cost Wearable Sensors for Healthcare Monitoring

**DOI:** 10.3390/mi16070791

**Published:** 2025-07-02

**Authors:** Tatiana Pereira Filgueiras, Pedro Bertemes-Filho, Fabrício Noveletto

**Affiliations:** Department of Electrical Engineering, Santa Catarina State University, Joinville 89219-710, SC, Brazil; pedro.bertemes@udesc.br (P.B.-F.); fabricio.noveletto@udesc.br (F.N.)

**Keywords:** wearable sensors, health monitoring, low-cost devices, photoplethysmography, biomedical sensor accuracy

## Abstract

This study evaluates the accuracy of a low-cost wearable system for the continuous monitoring of vital signs, including heart rate, blood oxygen saturation (Sp$O_{2}$), blood pressure trend (BPT), and body temperature. The prototype was built using the nRF52840 microcontroller, which integrates photoplethysmography and infrared sensors. The heart rate and Sp$O_{2}$ data were collected under three body positions (*Rest*, *Sitting*, and *Standing*), while all measurements were performed using both anatomical configurations: BPT-Finger and BPT-Earlobe. Results were compared against validated commercial devices: UT-100 for heart rate and Sp$O_{2}$, G-TECH LA800 for blood pressure, and G-TECH THGTSC3 for body temperature. Ten participants were monitored over a ten-day period. Bland–Altman analysis revealed clinically acceptable agreement thresholds of ±5 mmHg for blood pressure, ±5–10 bpm for heart rate, ±4% for Sp$O_{2}$, and ±0.5 °C for temperature. Both wearable configurations demonstrated clinically acceptable agreement across all vital signs. The BPT-Earlobe configuration exhibited superior stability and lower variability in the *Rest* and *Sitting* positions, likely due to reduced motion artifacts. Conversely, the BPT-Finger configuration showed higher Sp$O_{2}$ accuracy in the *Standing* position, with narrower limits of agreement. These findings highlight the importance of sensor placement in maintaining measurement consistency across physiological conditions. With an estimated cost of only ~USD 130—compared to ~USD 590 for the commercial alternatives—the proposed system presents a cost-effective, scalable, and accessible solution for decentralized health monitoring, particularly in underserved or remote environments.

## 1. Introduction

The use of microcontroller-based module sensors in healthcare IoT systems has been the subject of extensive research in recent years. The development of wearable devices using a 32-bit ARM® Cortex-M4 processor (MCU nRF52840) with Arm® Mbed™ OS for health monitoring has received significant research efforts, as they enable the continuous tracking of vital signs and the early detection of potential health issues [1]. Smart sensor systems, in particular, have shown great potential in this domain, with the capability to capture and wirelessly transmit a wide range of physiological data [2]. The precision and reliability of sensors are essential to ensure that the collected data is both useful and secure [3]. Moreover, the wearable solution is not only capable of mobile and real-time monitoring but also benefits from a low production cost and ergonomic design, making it accessible for broader populations and suitable for use in remote or resource-constrained environments. The combination of mobility, wearability, and affordability—achieved with an estimated prototype cost of only USD 130 compared to USD 590 for commercial devices—represents a significant advantage in promoting equitable access to digital health technologies.

In addition to healthcare applications, wearable sensor technologies have also been utilized in athletic and industrial settings, enabling real-time monitoring of physical activity, vital signs, and various other parameters that can provide valuable insights and enable data-driven decision-making [4].

When selecting sensors for healthcare IoT systems, balancing the cost-effectiveness of commercial sensors with the customizability and flexibility of MCU-based solutions is essential. Commercial sensors typically benefit from rigorous quality control and calibration processes, ensuring a high degree of precision and reliability [5]. In contrast, module sensors provide a more affordable and flexible approach, allowing for the integration of custom sensing capabilities and the exploration of novel applications [6].

Regarding the accuracy of module sensors, some studies demonstrate that the data collected by these sensors are comparable in accuracy to commercial devices, with the added benefit of faster data acquisition and storage, allowing for quicker interventions [7,8,9,10]. These studies argue that, when properly calibrated, module sensors can provide reliable data, especially when compared to more expensive devices, and in many cases, the differences fall within acceptable limits.

This paper presents a comparative analysis of sensor precision in MCU-based modules for healthcare monitoring. Our proposal evaluates the feasibility of using these sensors as an alternative to commercially available medical devices approved by regulatory bodies in Brazil, such as ANVISA [11] and INMETRO [12]. This study also assesses the accuracy and reliability of module sensors and investigates their potential applicability as a biomedical monitoring system.

## 2. Materials and Methods

This study was approved by the Ethics Committee for Research with Humans of the State University of Santa Catarina (CAAE 82997424.7.0000.0118). A wearable device was developed based on the nRF52840 MCU, incorporating a photoplethysmography sensor for measuring Sp$O_{2}$, heart rate, and blood pressure trend (BPT)—an estimate of blood pressure variation over time, making it useful for identifying hypertensive or hypotensive tendencies—as well as an infrared sensor for body temperature measurement. The prototype was designed to simultaneously monitor these vital signs. It was intended to be worn on the arm or forearm, with the possibility of incorporating additional functionalities in future iterations, such as fall detection and geolocation.

**Figure 1:**
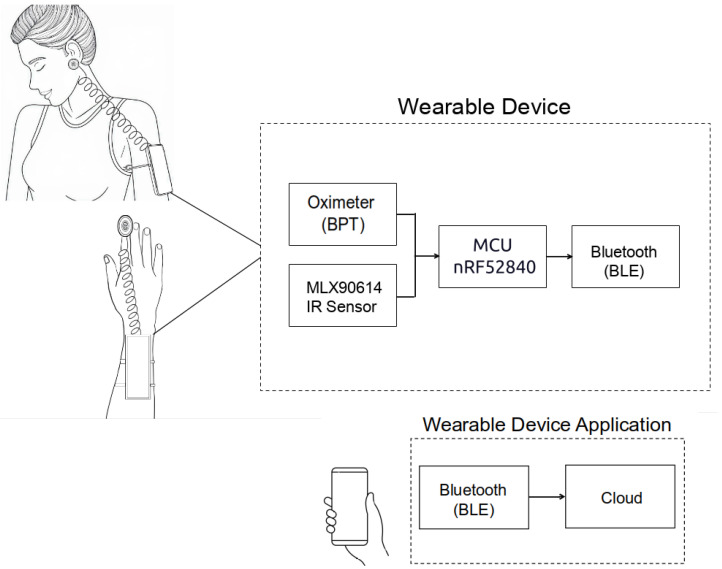
Wearable prototype in both anatomical configurations (BPT-Earlobe and BPT-Finger), along with the mobile application interface.

The experiment involved ten participants (Table 1) over ten days with one measurement taken per day. However, these were not consecutive days, as data collection sessions were scheduled according to participant availability. The device was tested on two anatomical sites: the earlobe (BPT-Earlobe) and the index finger (BPT-Finger) (Figure 1). On the earlobe, the wearable device was secured to the participant’s arm with an extension reaching the ear. For index finger measurements, the prototype was positioned on the wrist like a bracelet, with an extension resembling a thimble.

**Figure 2:**
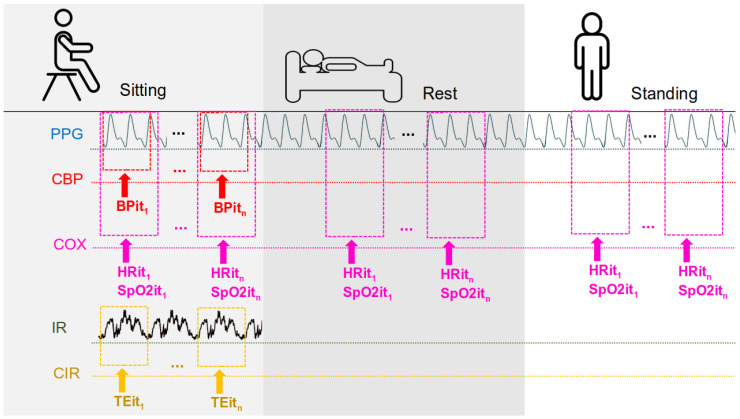
Measurements according to the participant’s position are as follows: PPG from this study; CBP—commercial blood pressure (commercial device); COX—commercial oximeter; IR—IR thermometer sensor from this work; CIR—commercial IR thermometer; BPi$t_{1-n}$—blood pressure from iteration 1 to *n*; HRi$t_{1-n}$—heart rate from iteration 1 to *n*; Sp$O_{2}$i$t_{1-n}$—Sp$O_{2}$ from iteration 1 to *n*; and TEi$t_{1-n}$—temperature from iteration 1 to *n*.

Three body postures were evaluated for the measurement of heart rate and Sp$O_{2}$—*Rest*, *Sitting*, and *Standing*—in order to encompass typical physiological variations encountered in daily life (Figure 2). Postural changes lead to significant alterations in hemodynamic and peripheral perfusion due to the effects of gravity and autonomic regulation [13]. For instance, transitioning from a supine to an upright position causes the redistribution of blood volume and a reflex-mediated increase in heart rate to maintain blood pressure, while the supine position promotes greater venous return and modifies perfusion in the extremities [13,14]. Such differences directly influence the vital signs measured by the sensors: studies have demonstrated variations in parameters such as oxygen saturation and skin temperature depending on posture—with better oxygenation observed in the upright Sitting position compared to the supine position and differences in skin temperature between lying and Standing due to the redistribution of peripheral blood flow [14,15,16]. Therefore, evaluating the device in all three postures ensures that its performance is tested under distinct physiological conditions (from Rest to orthostatic stress), thereby enhancing the ecological validity of the experiment.

Measurements were conducted simultaneously to ensure experimental consistency. The UT-100 pulse oximeter was placed on the finger of one hand, while the wearable device was worn on the opposite hand’s finger. Additionally, another configuration was tested, where the UT-100 was placed on a finger, while the prototype’s oximeter measured from the earlobe. This setup allowed for comparative validation between anatomical sites.

Each position previously mentioned was measured separately at different times throughout the same day to prevent potential bias or adaptation effects. All measurements were conducted using the same device for each participant to ensure consistency. For each measurement session, participants first remained in a seated position for at least 5 min before data collection (*Sitting*). Later in the day, they lay down for at least 3 min before measurements were taken in the *Rest* position. Finally, in a separate session, participants stood upright for at least 2 min before the *Standing* measurements were recorded. This sequential approach ensured that physiological changes associated with posture transitions did not interfere with the accuracy of the recorded data.

Blood pressure and temperature measurements were taken only in the *Sitting* position, following the same procedure. Temperature readings were collected from the participant’s arm using both the wearable device and a commercial thermometer.

To avoid measurement interference, the wearable device was always used before the commercial sphygmomanometer, as cuff inflation could affect the MCU sensor readings. All other measurements adhered to standardized protocols, with blood pressure and estimated BPT values recorded from each participant’s left arm and left earlobe [17,18].

### 2.1. Wearable Measuring System

The developed device is powered by the nRF52840 microcontroller from Nordic Semiconductor (Trondheim, Noruega), featuring a 32-bit ARM Cortex-M4 architecture operating at 64 MHz, with built-in capabilities for AI processing. These specifications make it well-suited for future embedded intelligence applications. The board includes 256 KB of SRAM and 1 MB of flash memory. It also supports Bluetooth^®^ 5 and Bluetooth Low Energy (BLE), which are employed in this project to transmit participant data to a custom-developed smartphone application, allowing users to monitor signals received from the sensor modules.

Concerning the oximeter, the PC-MED-0411 module, based on the MAX32664 sensor (Analog Devices, Wilmington, MA, USA) hub’s integrated circuit, was chosen. Through this module, it is possible to continuously measure estimated blood pressure (BPT—blood pressure trend) using photoplethysmography (PPG) to detect changes in blood volume in the microvascular bed of tissues, as well as to measure heart rate and Sp$O_{2}$ using the integrated MAX30102 sensor (Analog Devices, Wilmington, MA, USA).

The MLX90614 infrared sensor (Melexis, Shangai, China), a non-contact digital thermopile, was used for body temperature measurements. This sensor provides precise temperature readings without the need for direct contact with the body.

**Figure 3:**
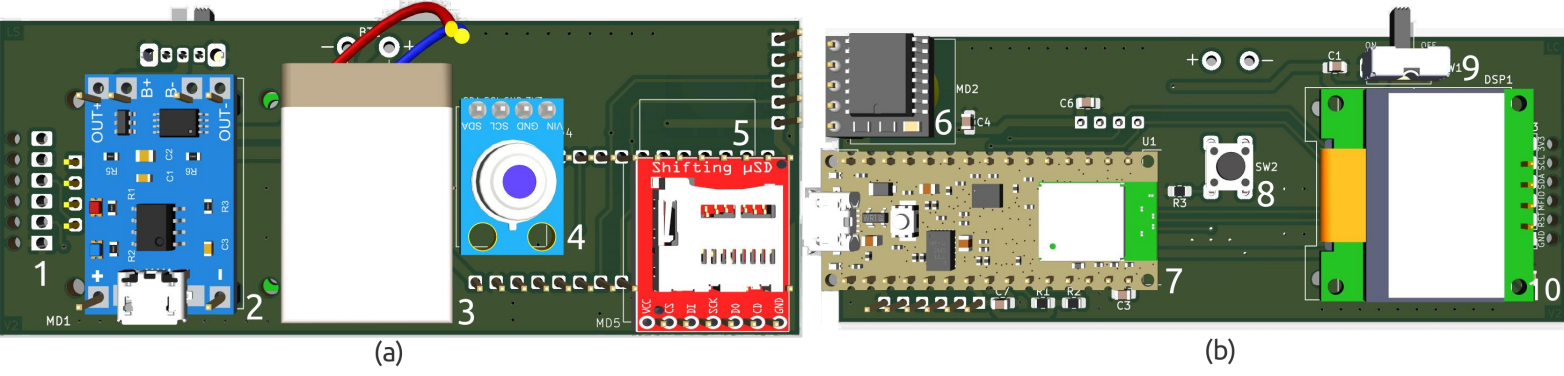
Three-dimensional schematic diagrams of the customized wearable device. (**a**) Rear view of the board, showing the 6-pin interface of the MAX32664 module (1), the power management module (2), the battery (3), the infrared (IR) sensor (4), and the microSD card interface (5). (**b**) Front view of the board, including the real-time clock (RTC) (6), the nRF52840 microcontroller (7), the button to reactivate the display from standby mode (8), the on/off power switch (9), and the display (10).

The total estimated cost of the prototype, including the microcontroller, sensor modules, and housing components, was approximately USD 130. The hardware of the prototype is shown in Figure 3, which presents a 3D view of the printed circuit board (PCB) without the MAX32664 sensor module. The connector of this module is a 6-pin interface placed on the left side of the board, as can be seen in Figure 3a.

### 2.2. Commercial Measuring System

As discussed in the Section 1, the reference devices were chosen for their proven accuracy (evaluated by regulatory agencies) and their widespread use in Brazil. For blood oxygen saturation and heart rate measurements, we used the UT−100 fingertip pulse oximeter (UTECH Co.,Ltd, Chongqing, China). This device provided reference Sp$O_{2}$ and pulse readings for each participant. For blood pressure and body temperature measurements, we selected two G-TECH devices known for professional-grade performance at an affordable cost. Blood pressure was measured with the G-TECH LA800 arm cuff sphygmomanometer(G-TECH Saúde, Rio de Janeiro, Brazil), and body temperature was measured with the G-TECH THGTSC3 infrared thermometer (G-TECH Saúde, Rio de Janeiro, Brazil). The combined cost of these three commercial reference devices is approximately USD 590 (UT-100: USD 400, LA800: USD 120, and THGTSC3: USD 70).

### 2.3. Acceptable Measurement Error Thresholds

To evaluate the accuracy of the prototype, we defined acceptable error margins based on established standards in the literature. For blood pressure, an error margin of up to 5 mmHg is considered acceptable in both clinical and monitoring contexts [19,20,21,22,23,24,25]. For heart rate, a deviation of up to 5% is typically acceptable for clinical use, with some contexts allowing up to 10% [26,27,28,29,30,31]. For oxygen saturation (Sp$O_{2}$), a deviation of up to 4% is widely accepted in clinical applications, including both medical-grade devices and wearable pulse oximeters such as the Garmin Fenix 5X Plus (Garmin Ltd., Chicago, IL, USA) and Apple Watch [32,33,34,35,36,37,38]. Regarding infrared thermometry, particularly measurements taken via the tympanic membrane and temporal artery, the literature indicates that an acceptable error margin is ±0.5 °C compared to standard reference methods (e.g., rectal and esophageal temperatures). This level of accuracy establishes infrared thermometry as a reliable alternative for fever detection across various populations [39,40,41,42,43].

By considering these acceptable error thresholds, this study aims to determine whether the wearable sensors in device meets the accuracy standards required for reliable health monitoring.

### 2.4. Software

**Figure 4:**
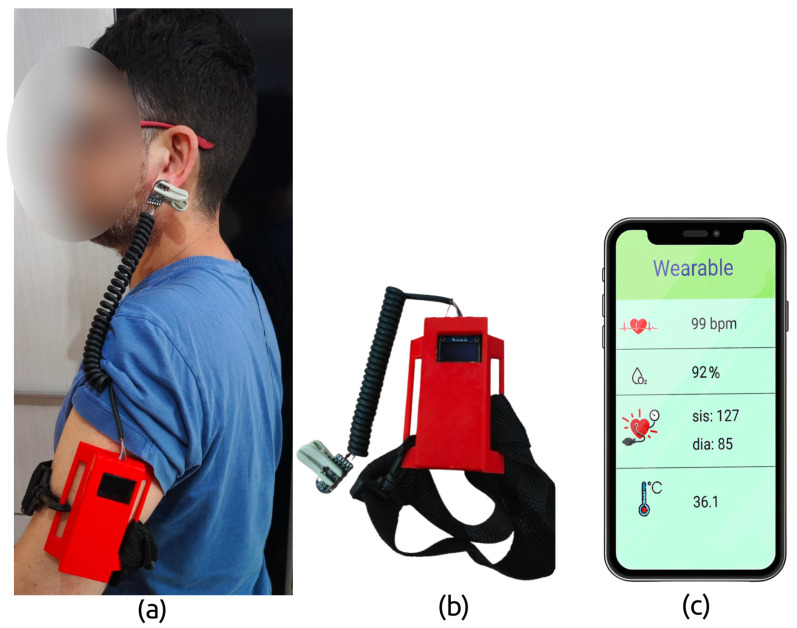
Prototype of the wearable device developed in this study: (**a**) the wearable system in use, showing the BPT-Earlobe configuration; (**b**) the wearable prototype and attachment strap; and (**c**) the graphical interface of the custom mobile application.

The firmware for the wearable was developed in C using the GNU GCC toolchain and deployed on the nRF52840 MCU running Arm^®^ Mbed™ OS. The microcontroller continuously monitors incoming signals from the PPG and IR sensor modules. A Bluetooth Low Energy (BLE) module transmits the data in text format to a custom smartphone application in real time. The mobile app (developed with Flutter for cross-platform Android/iOS support) displays the vital sign data and also uploads it to a cloud server via MQTT in the background, which helps conserve the wearable’s battery. BLE communication ensures that the user can move freely while data is being recorded and sent to the cloud for storage or further analysis. The prototype and the app interface are illustrated in Figure 4.

## 3. Results

Table 2 and Table 3 present, respectively, heart rate and oxygen saturation (Sp$O_{2}$) measurements conducted in the three previously mentioned positions: *Sitting*, *Rest*, and *Standing*. Table columns display values from the reference UT-100 device and the wearable prototype, configured as BPT-Finger and BPT-Earlobe, and display the mean values of the measurements taken over ten days. Additionally, the columns “Error Rate BPT-Earlobe” and “Error Rate BPT-Finger” represent the minimum and maximum values of the absolute percentage error of the measurements taken by the prototype in comparison to the values obtained by the UT-100 device.

Table 4 presents the mean values and standard deviations of the temperatures recorded over ten days for each participant. The columns include the results obtained from the commercial device G-TECH THGTSC3, used as a reference, and the IR temperature sensor MLX90614, integrated into the prototype developed for this study. Additionally, the minimum and maximum absolute error values, measured in degrees Celsius, relative to the data provided by the G-TECH THGTSC3 device, are reported.

Table 5 presents the mean values and standard deviations of blood pressure and estimated blood pressure measurements recorded over ten days for each participant. The columns include the results obtained from the commercial device GTECH LA800, used as a reference, and the prototype in its BPT-Finger and BPT-Earlobe configurations. Additionally, the minimum and maximum absolute error values, expressed in mmHg and calculated relative to the data provided by the GTECH LA800 device, are presented. In the “Error Rate” column, the subcolumns “Finger” and “Earlobe” indicate the prototype’s maximum absolute error in the BPT-Finger and BPT-Earlobe configurations, respectively.

## 4. Discussions

**Figure 5:**
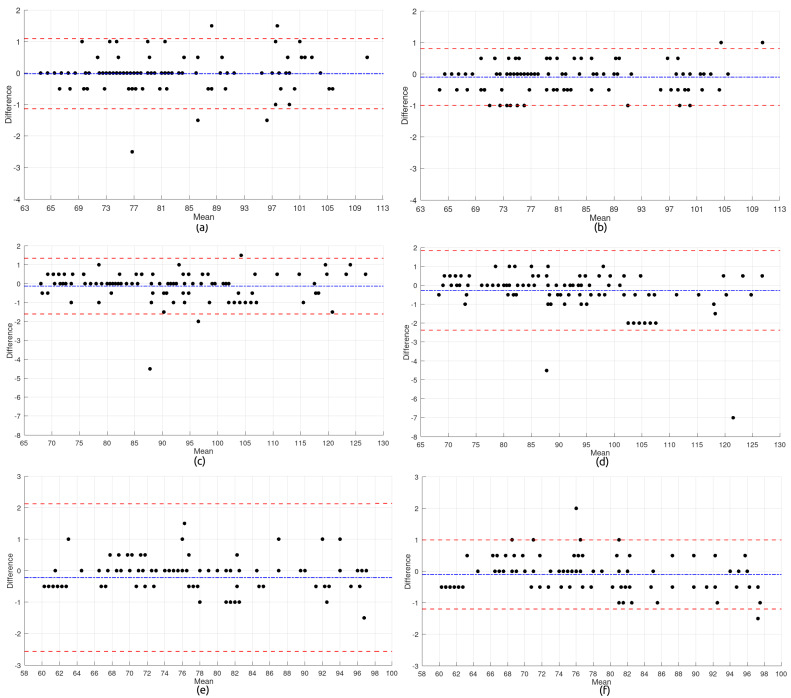
Heart rate subfigures: (**a**) BPT-Finger—Sitting; (**b**) BPT-Earlobe—Sitting; (**c**) BPT-Finger—Standing; (**d**) BPT-Earlobe—Standing; (**e**) BPT-Finger—Rest; and (**f**) BPT-Earlobe—Rest.

Beyond assessing sensor performance, this study also investigated whether a low-cost alternative—namely, the USD 130 prototype—could deliver measurement reliability comparable to commercial systems priced over four times higher. The agreement between heart rate values recorded by the UT-100 device and the proposed solution was examined through Bland–Altman analysis (Figure 5). These plots visualize the differences between the two devices (UT-100 minus the prototype) as a function of their mean, allowing for the evaluation of both bias and dispersion across body positions and sensor placements.

In each plot, the central blue dashed line represents the average difference (bias), while the upper and lower red dashed lines indicate the limits of agreement (LoAs), defined as the bias ±1.96 times the standard deviation of the differences. The following equations were used to compute these values:

(1) $$\mathrm{Bias}=\bar{d}=\frac{1}{n}\sum_{i=1}^{n}\left(x_{i}-y_{i}\right)$$

(2) $$\mathrm{SD}=\sqrt{\frac{1}{n-1}\sum_{i=1}^{n}{\left(\left(x_{i}-y_{i}\right)-\bar{d}\right)}^{2}}$$

(3) $$\mathrm{Upper}\;\mathrm{Limit}\;\mathrm{of}\;\mathrm{Agreement}\;\left(\mathrm{ULA}\right)=\bar{d}+1.96\times \mathrm{SD}$$

(4) $$\mathrm{Lower}\;\mathrm{Limit}\;\mathrm{of}\;\mathrm{Agreement}\;\left(\mathrm{LLA}\right)=\bar{d}-1.96\times \mathrm{SD}$$

where

- $x_{i}$ and $y_{i}$ are the measurements obtained from the two methods for the same subject *i*;
- $\bar{d}$ is the mean difference between methods;
- SD is the standard deviation of the differences;
- The factor 1.96 assumes that the differences follow a normal distribution, corresponding to a 95% confidence interval.

Subfigures (a) through (f) correspond to the comparisons between the commercial and wearable devices in each anatomical configuration (BPT-Finger and BPT-Earlobe) and body posture (*Sitting*, *Standing*, and *Rest*).

In the *Sitting* posture, BPT-Finger measurements showed a bias near zero, with most values clustered between −1 and +1 bpm, indicating satisfactory agreement. A few outliers were present, reaching up to ±2 bpm, but the overall distribution was homogeneous, with no signs of heteroscedasticity—highlighting the configuration’s stability under these conditions. BPT-Earlobe, however, exhibited even tighter dispersion and minimal noise, with virtually all data points falling within ±1 bpm and narrower limits of agreement, emerging as the most precise setup in this posture. The superior performance of the BPT-Earlobe configuration can be partially attributed to its reduced susceptibility to motion artifacts. Anatomically, the earlobe presents fewer voluntary and involuntary movements compared to the finger, especially during Sitting and resting postures. This relative stability minimizes external mechanical interferences, allowing a more stable photoplethysmographic (PPG) signal. Additionally, the earlobe has a relatively consistent vascular structure with lower peripheral vasoconstriction, which allows for a more stable perfusion and, consequently, higher signal quality. These characteristics are particularly beneficial in low-motion contexts, where minor hand tremors or digit movements can compromise the signal integrity in finger-based configurations.

The *Standing* posture introduced greater variability. BPT-Finger results revealed increased dispersion, with deviations surpassing ±4 bpm and outliers reaching −5 bpm, although the average bias remained low. This degradation in accuracy is likely due to reduced peripheral perfusion in the upright position. In contrast, BPT-Earlobe data were more consistent, although some asymmetry in limits of agreement and underestimation at higher heart rates (>100 bpm) suggested possible heteroscedasticity and signal artifacts under orthostatic stress.

During the *Rest* condition, the BPT-Finger configuration demonstrated the weakest performance. Outliers reached −10 bpm, with positive bias and a broad dispersion range, indicating overestimation and poor reliability. These results may be influenced by peripheral perfusion changes associated with the supine posture. Meanwhile, the BPT-Earlobe configuration performed better under these conditions, with moderate spread, bias close to zero, and most values confined within ±1 bpm—highlighting its robustness even when perfusion is potentially compromised.

The overall analysis of the six Bland–Altman plots yields valuable findings for both clinical implementation and future sensor design:

- **Influence of Body Posture:** Measurement consistency was significantly affected by participant position. The *Standing* posture led to the highest variability, particularly in the BPT-Earlobe setup, where the most extreme outliers (up to −7 bpm) were found. In contrast, the *Sitting* position provided the most stable environment for both configurations.
- **Sensor Configuration Accuracy:** While the BPT-Finger configuration exhibited wider agreement limits—especially in the *Rest* condition—BPT-Earlobe showed greater fluctuation in the *Standing* posture. Moreover, the earlobe setup often presented discretized readings, suggesting possible limitations in digital resolution or processing precision.
- **Outliers and Noise:** Significant outliers, such as −11 bpm for BPT-Finger at *Rest* and −7 bpm for BPT-Earlobe at *Standing*, highlight susceptibility to motion or perfusion-related artifacts. These findings underscore the need for improved artifact detection and correction algorithms.
- **Signal Distribution Patterns:** The recurring trimodal distribution observed in BPT-Earlobe measurements may indicate a systemic feature of the signal acquisition process, potentially related to quantization or internal filtering—warranting further technical investigation.

**Figure 6:**
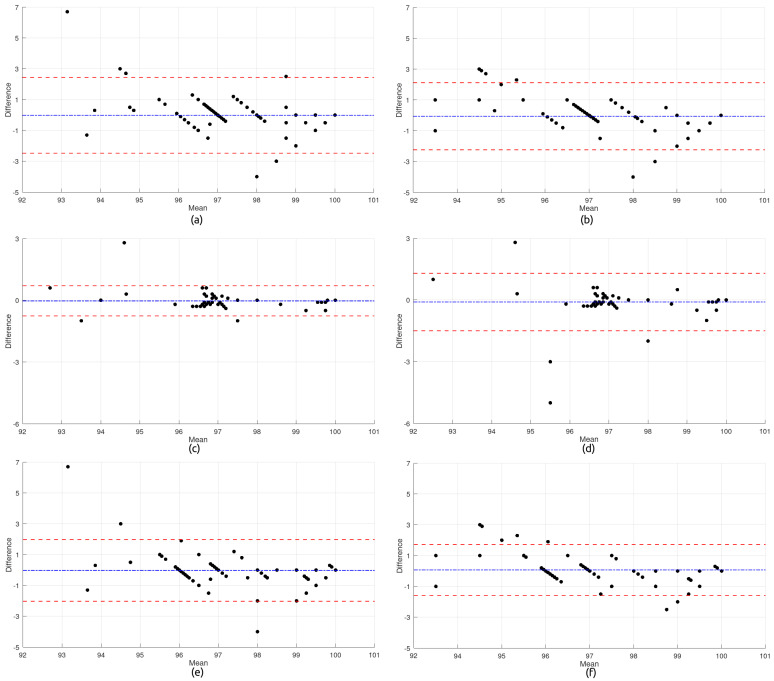
Sp$O_{2}$ subfigures: (**a**) BPT-Finger—Sitting; (**b**) BPT-Earlobe—Sitting; (**c**) BPT-Finger—Standing; (**d**) BPT-Earlobe—Standing; (**e**) BPT-Finger—Rest; and (**f**) BPT-Earlobe—Rest.

The plots presented in Figure 6 provide a comprehensive comparative analysis between the wearable device developed in this study, in both the BPT-Finger and BPT-Earlobe configurations, and the commercial reference device UT-100 for oxygen saturation (Sp$O_{2}$) measurements. This analysis was also conducted across three different body positions (*Sitting*, *Standing*, and *Rest*).

Subfigures (a) and (b) present, respectively, the metrics of the BPT-Finger and BPT-Earlobe configurations compared to the UT-100 device in the *Sitting* position. Subfigures (c) and (d) show the same comparisons in the *Standing* position. Lastly, subfigures (e) and (f) display the metrics for the BPT-Finger and BPT-Earlobe configurations, respectively, in comparison to the UT-100 device in the *Rest* position.

In the *Sitting* position, the BPT-Finger configuration exhibited a slight tendency to underestimate Sp$O_{2}$ values compared to the UT-100 device, with a slightly negative mean difference. The limits of agreement ranged approximately from +2.5% to −2.5%, with most measurements concentrated between 0% and −1%. An interesting clustering pattern was observed in specific ranges (96–97% and 97–98%), suggesting a possible quantization effect in the readings. Notably, two significant outliers were detected: one extreme positive value near +6.5% (in the 93% Sp$O_{2}$ range) and one negative value close to −4% (around 98% Sp$O_{2}$).

The BPT-Earlobe configuration in the same position showed greater variability, with limits of agreement ranging from approximately +2.0% to −2.0%. The distribution revealed visible clustering along diagonal lines, suggesting a proportional bias in the measurements—i.e., the higher the average Sp$O_{2}$ value, the greater the tendency of the BPT-Earlobe device to underestimate. Significant outliers were observed around −4%, along with several points above +2%, including readings close to +3% in the 94–95% Sp$O_{2}$ range.

In the *Standing* position, the BPT-Finger configuration exhibited the lowest variability among all tested configurations, with the narrowest limits of agreement, approximately between +0.8% and −0.8%. The distribution was highly concentrated around values close to 0% and −0.3%, particularly in the 96–97% Sp$O_{2}$ range. One notable outlier appeared around +3% in the region of 94% Sp$O_{2}$.

The BPT-Earlobe configuration in the same position exhibited a distinct pattern, with most measurements clustered near a 0% difference, especially in the 96–97% Sp$O_{2}$ range. However, greater dispersion was observed in other regions, with significant outliers reaching −5% and +3%. The limits of agreement were approximately between +1.5% and −1.5%.

In the *Rest* position, the BPT-Finger configuration demonstrated behavior similar to that observed in the *Sitting* position, with limits of agreement between approximately +2.0% and −2.0%. Again, an extreme outlier of approximately +6.5% was observed in the 93% Sp$O_{2}$ range, suggesting potential systematic vulnerability of the device at lower saturation levels. Most measurements were concentrated around a 0% difference, with visible clustering in specific ranges.

The BPT-Earlobe configuration in the same position exhibited a more structured pattern, with measurements clearly grouped along discrete horizontal lines (approximately at +3%, +1%, 0%, −0.5%, and −1.5%). This pattern suggests limited digital resolution or quantization effects in the readings from this configuration. The limits of agreement were approximately between +1.8% and −1.5%.

The integrated analysis of the six Bland–Altman plots for Sp$O_{2}$ reveals important aspects for both clinical applications and technological development:

- **Effect of Body Position:** Body posture had a significant impact on the agreement of Sp$O_{2}$ measurements. Notably, the *Standing* position yielded the most consistent readings for the BPT-Finger configuration, while the *Sitting* position exhibited greater variability for both configurations. This finding contrasts with what is typically observed in heart rate measurements, where the resting position usually offers greater stability.
- **Comparison Between Configurations:** The BPT-Finger configuration generally showed limits of agreement similar to those of the BPT-Earlobe. However, it presented a greater occurrence of extreme outliers at lower Sp$O_{2}$ values (around 93%). The BPT-Earlobe configuration, in turn, exhibited more evident quantization patterns, particularly in the *Rest* position.
- **Accuracy Across Different Sp$O_{2}$ Ranges:** Both configurations demonstrated better agreement with the reference device at Sp$O_{2}$ levels ranging from 97% to 99%, which correspond to normal oxygen saturation in healthy adults. However, greater variability and the presence of outliers were observed in lower ranges (93–95%), which is critical in clinical applications where the detection of moderate hypoxemia is essential.
- **Quantization Patterns:** The distinct clustering patterns observed—particularly in the BPT-Earlobe configuration during the *Rest* position—suggest limitations in digital resolution or in the signal processing algorithm. This “stepping” effect in the measurements may impair the device’s ability to detect subtle changes in oxygen saturation.

These findings lead to the conclusion that both configurations of the prototype demonstrated clinically acceptable agreement with the commercial UT-100 device for Sp$O_{2}$ monitoring in healthy individuals, with most measurements falling within the ±2% margin across all three tested positions. This level of agreement is consistent with international guidelines [32,33,34,35,36,37,38].

Among the configurations evaluated, the BPT-Finger configuration in the *Standing* position showed the best overall performance, standing out due to its narrower limits of agreement and lower dispersion in values, making it the preferred option for spot-check Sp$O_{2}$ measurements. On the other hand, the BPT-Earlobe configuration exhibited more noticeable quantization patterns, especially in the *Rest* position, suggesting the need for improvements in signal processing for this configuration. Nevertheless, its overall agreement remained within clinically acceptable thresholds, offering a viable alternative in situations where the BPT-Finger configuration is not practical or comfortable for the user.

The presence of significant outliers at lower Sp$O_{2}$ values (93–94%) indicates that caution is warranted when interpreting readings in this range, particularly in clinical contexts where the early detection of hypoxemia is critical. In such cases, it is recommended that additional validation be performed using invasive reference measurements, especially for patients with respiratory compromise.

Furthermore, the proportional bias patterns observed—particularly with the BPT-Earlobe configuration in the *Sitting* position—highlight the importance of implementing calibration algorithms tailored to different saturation ranges. This could enhance the device’s accuracy across the full clinically relevant spectrum of Sp$O_{2}$ values.

**Figure 7:**
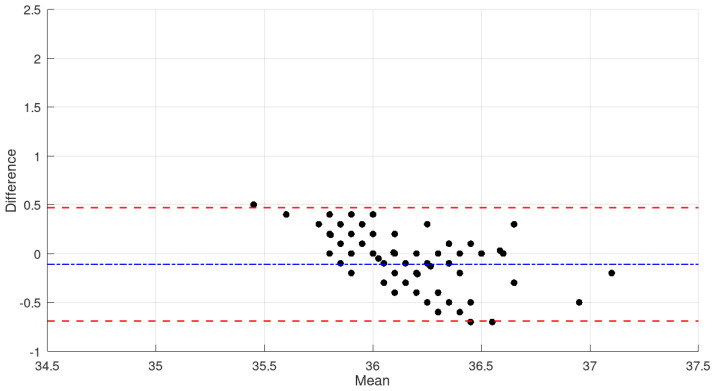
Comparison of the mean temperature values measured in all subjects by the G-TECH THGTSC3 device and the prototype sensor.

The infrared detection employed by the IR MLX90614 sensor is particularly sensitive to external interferences [44]. The graph in Figure 7 presents the evaluation of the reliability of the proposed wearable device by comparing it to the commercial infrared thermometer GTECH.

The analysis of the graph shows that the mean difference between the measurements obtained from the proposed wearable device and the GTECH thermometer is approximately −0.1 °C (blue dashed line), indicating a slight tendency of the wearable device to produce lower readings compared to the commercial reference. The limits of agreement (red dashed lines) range approximately from −0.8 °C to +0.6 °C, demonstrating that 95% of the differences fall within this interval.

It is important to note that most data points are randomly distributed around the mean, with no evident systematic patterns, suggesting the absence of proportional bias. However, a single outlier near 34.9 °C with a difference of approximately +2.0 °C was observed, which may represent an isolated measurement during the sensor’s stabilization phase or the result of transient environmental conditions.

For applications involving the continuous monitoring of body temperature, the limits of agreement observed in this study may be considered clinically acceptable, given that variations up to ±0.5 °C are often tolerated in screening devices [39,40,41,42,43]. The mean difference close to zero indicates good calibration of the developed device in relation to the commercial standard.

A subtle trend of decreasing differences as the average temperature increases, particularly in the 36.0 °C to 36.5 °C range, was also observed. This suggests improved performance of the MLX90614 sensor at temperatures closer to human normothermia. Such a characteristic is advantageous for the intended application, in which the accurate detection of febrile states is essential.

The dispersion of differences within the 35.5 °C to 37.1 °C range demonstrates that the wearable device maintains satisfactory consistency across the clinically relevant temperature range, supporting its applicability as a non-invasive tool for continuous temperature monitoring.

**Figure 8:**
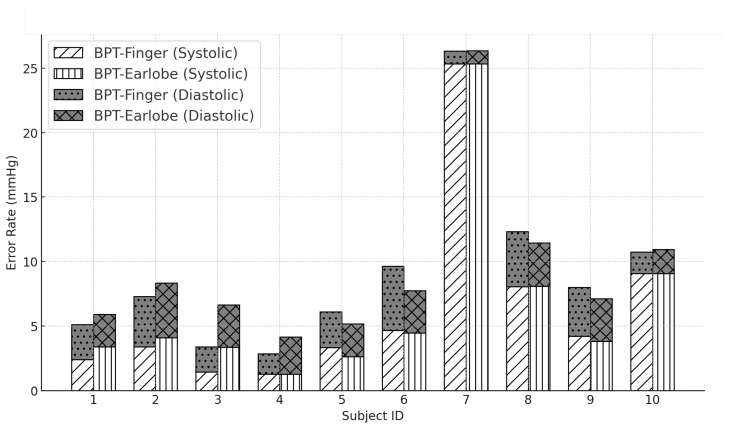
Comparison of error rates for the BPT-Finger and BPT-Earlobe configurations relative to the mean systolic and diastolic blood pressure values measured by the commercial device GTECH LA800 in the participants of this study.

As aforementioned in Section 1 of this study, the literature indicates that differences of up to 5 mmHg between blood pressure measurement devices are considered acceptable [19,20,21,22,23,24,25]. As shown in Table 5 and Figure 8, in most participants, the absolute error differences between the reference device and the estimated blood pressure sensor remained within this limit, demonstrating the feasibility of using the plethysmography-based approach in health monitoring contexts.

However, three participants (Subjects 7, 8, and 10) exhibited errors greater than 5 mmHg in the measurements. Subject 7, in particular, showed differences in systolic pressure of approximately 25 mmHg in both prototype configurations, which may be explained by their history of hypertension. Implementing personalized sensor calibration for hypertensive individuals could reduce these discrepancies. A similar issue was observed in Subject 8, who also has a history of hypertension and showed significantly altered values.

As for Subject 10, although they had no history of hypertension, they reported experiencing increased stress at work and at home during the measurement period, which may have contributed to episodes of elevated blood pressure observed in this participant [45].

Another possible cause of these discrepancies could be the nature of plethysmography, which is influenced by external factors such as peripheral blood flow, ambient temperature, and motion artifacts [46]. Unlike the GTECH LA800 device, which uses the gold-standard cuff inflation method, plethysmographic sensors estimate blood pressure based on changes in tissue blood volume, making them more susceptible to external variations.

Based on the data presented, the BPT-Earlobe configuration demonstrated slightly better performance in terms of precision and consistency when compared to the BPT-Finger configuration. This behavior can be attributed to factors such as the reduced impact of motion on the earlobe, which tends to be less susceptible to movement artifacts than the index finger.

The analysis of error rates revealed that, in most cases, the readings from the BPT-Earlobe configuration were equal to or lower than those recorded with BPT-Finger. For instance, although both configurations showed significant discrepancies in participants with a history of hypertension, BPT-Earlobe demonstrated greater stability and smaller variations across the remainder of the sample when compared to the reference device (GTECH LA800). Moreover, in the *Rest* and *Sitting* positions, BPT-Earlobe provided more consistent readings, while BPT-Finger exhibited greater sensitivity to variations caused by peripheral blood flow and finger movements. This difference is particularly relevant in dynamic monitoring scenarios, where the stability of measurements may be compromised. This trend was also evident in the Sp$O_{2}$ and heart rate recordings.

The work contributes with a comprehensive analysis of sensor accuracy and also investigates the practical viability of the device in real-world scenarios. Since it is wearable and has a low cost, these features might significantly enhance its use in home care settings, remote areas, and low-income populations with limited access to medical equipment. Additionally, the portability enables real-time and dynamic monitoring without restricting the patient’s mobility. These features are especially relevant for elderly individuals in nursing homes or patients with chronic conditions requiring continuous supervision. By comparing this work with a more robust commercial system, the proposed device represents a meaningful advancement toward the democratization of digital health.

Furthermore, the device showed a consistent performance throughout the entire data acquisition period, without presenting any malfunctions or instability over time. It was powered by a lithium-ion polymer battery of 3.7 V and 500 mAh (model Liter Energy SD 502535), which sustained continuous monitoring for approximately 12 to 14 h on a single charge. This autonomy aligns well with a typical daily use scenario, requiring only overnight recharging. Based on the estimated energy consumption of the embedded components (the nRF52840 MCU, photoplethysmography, and infrared sensors), the average current draw was approximately 35 to 40 mA during operation. These values provide a practical reference for evaluating the device’s usability in real-world continuous monitoring applications.

One important distinction between the proposed device and commercial solutions lies in the sensitivity and signal processing capabilities of the sensor subsystem. The sensor’s module integrates the MAX30102 optical sensor with the MAX32664D biometric sensor hub, resulting in an enhanced performance for the detection of subtle physiological changes. The MAX30102 device features an 18-bit analog-to-digital converter (ADC), which offers significantly higher resolution than typical commercial devices such as the UT100 device, which operates with a functional resolution of approximately 1%. This higher resolution allows for the detection of minute variations in the reflected optical signal, enabling greater sensitivity in monitoring blood oxygen saturation (SpO_2_) and heart rate (HR).

While both systems support SpO_2_ readings from 0 to 100%, the UT−100 device specifies a measurement accuracy of ±2% in a range from 70 to 100%, with undefined accuracy below 70%. In contrast, the proposed solution applies embedded algorithms within the MAX32664D device to compensate for physiological noise, ambient light fluctuations, and motion artifacts, which can significantly degrade signal quality in wearable applications. These algorithms enhance reliability, particularly in dynamic scenarios, by using adaptive control of LED current and pulse width, as well as accelerometer-based motion compensation.

With respect to heart rate monitoring, both systems share the same operating range (30–250 bpm) and a nominal resolution of 1 bpm. However, the embedded algorithms in the MAX32664D device provide improved tolerance to movement and low-perfusion conditions. While the UT100 device states a tolerance of ±3% for SpO_2_ and ±3 bpm for HR under such scenarios, the MAX30102 device incorporates ambient light cancellation (ALC) and offers programmable acquisition parameters, further reinforced by the MAX32664D’s signal fusion techniques. These characteristics make the proposed device more robust and suitable for continuous, real-world wearable monitoring.

In terms of temperature sensing, the proposed device integrates the MLX90614 infrared sensor, which was compared to the G-TECH THGTSC3, a commercially available non-contact forehead thermometer. Both devices demonstrate comparable clinical accuracy, particularly within the physiological range relevant to human body temperature. The MLX90614 device presents an accuracy of ±0.2 °C in the range of 35 °C to 42 °C, which aligns with the typical performance of the G-TECH THGTSC3 of ±0.2 °C tolerance between 36 °C and 39 °C and ±0.3 °C outside that interval. A significant advantage of the MLX90614 lies in its high thermal resolution of 0.02 °C, which surpasses the 0.1 °C resolution offered by the THGTSC3. This finer granularity allows for the detection of small variations in body temperature, which is an important feature for the continuous monitoring and early detection of fever patterns.

Regarding measurement range, the MLX90614 supports a broader range from −70 °C to +380 °C for object temperature and −40 °C to +85 °C for ambient temperature. This make it suitable for diverse applications beyond human temperature monitoring. In contrast, the G-TECH THGTSC3 is limited to a narrower range of 32.0 °C to 42.9 °C, as it is calibrated specifically for human forehead readings.

Both sensors deliver a rapid response, typically within one second, and are intended for close-proximity use. While the G-TECH THGTSC3 is factory-calibrated and optimized for clinical forehead use at a fixed distance (typically 3–5 cm), the MLX90614 requires proper calibration and integration into a suitable housing for accurate and reliable medical measurements.

Although a minority of participants (=20%) reported some level of discomfort due to the reduced freedom of movement while wearing the device, the majority (=80%) expressed satisfaction with the convenience of monitoring their vital signs without the need for multiple instruments. Most participants preferred this system over conventional blood pressure monitors, emphasizing the absence of discomfort typically caused by cuff inflation. These insights reinforce the practical viability of the proposed solution for daily life applications.

### Study Limitations

One of the main limitations of this study lies in the selection of commercial devices as reference comparators. Although these instruments were chosen based on regulatory evaluations and widespread availability in the Brazilian market, they are not gold-standard tools typically employed in clinical validation protocols. Rather, they reflect devices commonly used in hospitals, clinics, and primary care settings, making them representative of real-world practice.

However, the absence of scientifically validated, research-grade reference equipment introduces potential bias in the comparison. While the selected commercial devices provide clinically accepted measurements, they may not offer the same level of accuracy and precision as laboratory-grade instruments. Consequently, observed discrepancies between the wearable prototype and the reference devices may partially stem from the inherent limitations of the latter.

It is also important to note that regulatory approval does not necessarily imply validation against gold-standard methodologies. Many commercially available devices are certified primarily for operational reliability and safety, rather than for strict clinical accuracy—especially under controlled, invasive, or laboratory settings. This factor should be taken into account when interpreting measurement differences reported in this study.

It must be emphasized that this work contains few limitations relate to the potential variability in sensor positioning and skin contact during repeated measurements. Although the device was consistently and precisely placed on both the left arm and earlobe by ensuring a constant contact pressure before each acquisition, small differences in strap tightness, angle, and skin condition may still play a parasitic interference role in the measured data. It is know that these subtle deviations in real-world wearable use may add fluctuations in signal quality. Therefore, future versions of the device should consider incorporating self-adjusting mechanical interfaces or adaptive contact surfaces to ensure more consistent signal acquisition in order to improve measurement reliability during long-term use.

Despite these constraints, the findings remain highly relevant for practical healthcare scenarios. Given that a substantial portion of patient monitoring is performed using cost-effective commercial devices, assessing the performance of wearable systems against such instruments provides meaningful insights into their feasibility and clinical utility. Nevertheless, future investigations should include validated clinical-grade reference equipment—such as hospital-certified pulse oximeters, sphygmomanometers, and infrared thermometers—to further substantiate the reliability of low-cost wearable solutions.

## 5. Conclusions

The findings of this study confirm that low-cost wearable sensors can achieve an acceptable reliability for continuous health monitoring, once it is properly validated. The prototype device based on the nRF52840 microcontroller showed good agreement with a commercial device for vital signs, such as heart rate, blood oxygen saturation (Sp$O_{2}$), estimated blood pressure, and body temperature.

Acoording to the Bland–Altman analysis, the wearable device maintained measurement discrepancies within both clinical and acceptable thresholds, which were ±5–10 bpm for heart rate, ±4% for SpO_2_, ±5 mmHg for blood pressure, and ±0.5 °C for temperature. These results validate the use of low-cost wearable sensors for multiparametric monitoring under dynamic physiological conditions.

The configuration positioned at the earlobe (BPT-Earlobe) exhibited superior stability and lower variability in both the *Rest* and *Sitting* positions, likely due to motion artifacts reduction and more consistent perfusion. In contrast, the BPT-Finger configuration was better during the *Standing* position for Sp$O_{2}$. The sensor placement is important for optimizing measurement accuracy under varying physiological and postural conditions.

It is important to clarify that the estimated cost of approximately USD 130 refers exclusively to the prototype’s hardware components (bill of materials) and does not account for expenses such as research and development, manufacturing, certification, or commercialization. The cost estimated is just for demonstrating the technical feasibility and potential accessibility of a modular, low-cost wearable system designed for continuous health monitoring—particularly in low-resource or remote settings.

Beyond the technical dimension, the use of energy-efficient and low-cost components sets this solution as a better social and environmental alternative in comparison to conventional monitoring technologies. Its adaptability and ease-to-use feature are especially valuable for telehealth contexts, low-income populations, and remote environments. Moreover, by enabling early detection and reducing the need for frequent in-person visits, this type of solution might be useful to reduce the environmental footprint in healthcare delivery.

While some limitations were observed under dynamic physiological conditions and individual variability, it can be concluded that the proposed wearable system is consistent and reliable across key clinical parameters and multiple body postures. Future work should focus on enhancing signal processing algorithms, incorporating personalized calibration strategies, and extending validation studies to include broader populations and disease profiles. These advancements will further solidify the role of low-cost wearable sensors as transformative tools in accessible, precision-driven digital health.

## Figures and Tables

**Table 1:** Summary of demographic and biometric data of the ten study participants.

Subject ID	Age	Height (cm)	BMI ^1^ (kg/m^2^)	Gender
1	40	171	39.30	Female
2	68	184	26.60	Male
3	74	156	27.20	Female
4	75	170	17.30	Female
5	60	185	25.27	Male
6	79	165	25.71	Female
7	87	155	17.78	Female
8	37	175	22.86	Female
9	48	166	29.03	Female
10	42	170	25.26	Male

^1^ Body mass index, defined as the weight (kg) divided by the square of the height (m^2^).

**Table 2:** Mean heart rate values measured in Subjects 1 to 10 and their respective absolute percentage error rates.

Position	Subject ID	UT-100 (bpm)	BPT-Finger (bpm)	BPT-Earlobe (bpm)	Error Rate BPT-Finger (%)	Error Rate BPT-Earlobe (%)
Sitting	1	100.95±4.82	101.10±4.78	101.05±4.54	0–1.57	0–1.02
	2	81.35±4.68	81.55±4.60	81.30±4.58	0–0.66	0–0.61
	3	99.30±3.97	99.15±3.85	99.65±3.73	0–0.99	0–1.10
	4	73.95±2.69	73.95±2.69	74.60±2.45	0–0.00	0–1.43
	5	80.90±6.62	81.00±6.46	80.75±6.68	0–1.49	0–1.38
	6	75.90±2.22	75.90±2.34	76.00±2.35	0–1.37	0–1.30
	7	66.70±2.57	66.75±2.60	66.80±2.53	0–0.74	0–0.79
	8	87.35±6.43	87.35±6.21	87.45±6.46	0–3.45	0–2.30
	9	74.00±4.27	73.80±4.05	73.90±4.14	0–1.35	0–0.76
	10	82.15±8.83	82.25±8.96	82.05±8.86	0–3.31	0–0.71
Rest	1	89.70±5.28	89.70±5.43	90.10±5.46	0–1.56	0–1.55
	2	78.90±7.27	78.95±7.57	78.80±7.30	0–1.57	0–1.23
	3	94.10±2.53	94.35±2.47	94.40±2.49	0–0.54	0–1.09
	4	61.45±0.83	61.95±0.83	61.95±0.83	0–0.83	0–0.83
	5	73.45±4.55	73.50±4.59	73.55±4.30	0–0.65	0–0.81
	6	73.00±3.33	73.00±3.26	72.90±3.45	0–0.75	0–0.75
	7	69.00±4.34	68.95±4.65	68.95±4.65	0–1.24	0–1.24
	8	79.50±4.85	79.45±5.07	79.45±5.20	0–1.95	0–2.60
	9	71.05±4.14	71.10±4.43	70.85±4.29	0–1.22	0–1.45
	10	77.45±5.82	78.90±7.49	77.70±6.11	0–1.35	0–1.40
Standing	1	119.70±4.19	120.20±3.46	120.55±3.33	0–4.82	0.39–4.82
	2	95.05±4.66	95.15±4.53	95.10±4.71	0–1.68	0–1.13
	3	105.50±4.83	106.20±4.63	106.90±4.58	0.45–1.02	0.43–1.97
	4	83.95±2.31	83.85±2.19	83.85±2.19	0–0.62	0–1.21
	5	91.30±6.24	91.20±5.75	91.35±5.91	0–1.43	0–1.14
	6	78.70±3.19	78.65±3.27	78.35±2.95	0–0.68	0–1.27
	7	71.45±3.03	71.15±2.78	71.25±2.83	0–1.27	0–1.38
	8	96.75±5.99	97.15±6.09	97.05±5.86	0–2.09	0–1.10
	9	76.35±8.09	76.80±8.60	76.80±8.61	0–5.26	0–5.26
	10	90.90±7.56	91.25±7.46	91.10±7.64	0–1.28	0–1.14

**Table 3:** Mean Sp$O_{2}$ values measured in Subjects 1 to 10 and their respective absolute percentage error rates.

Position	Subject ID	UT-100 (%)	BPT-Finger (%)	BPT-Earlobe (%)	Error Rate BPT-Finger (%)	Error Rate BPT-Earlobe (%)
Sitting	1	98.00±1.33	99.00±1.49	99.80±0.42	0–4.17	0–4.17
	2	95.11±3.14	95.91±3.61	95.70±2.78	0–3.45	0–2.17
	3	97.00±1.49	96.57±1.66	96.80±2.25	0–1.22	0–1.06
	4	97.00±0.05	96.94±0.12	96.92±0.09	0–0.10	0–0.10
	5	97.50±1.27	97.34±1.22	97.77±1.33	0–1.34	0–3.09
	6	96.80±1.03	96.81±1.05	96.81±1.05	0–0.32	0–0.32
	7	97.50±0.53	97.00±0.55	97.00±0.55	0–1.03	0–1.03
	8	97.60±1.26	97.84±2.19	97.84±2.19	0–3.09	0–3.09
	9	97.60±1.43	98.21±1.36	98.21±1.36	0–4.17	0–4.17
	10	97.50±1.78	97.70±2.06	97.69±2.06	0–4.17	0–4.17
Rest	1	97.80±1.48	98.70±1.49	98.40±2.07	0–3.10	0–3.10
	2	96.20±3.46	96.12±3.72	96.39±3.84	0–1.05	0–3.15
	3	96.10±1.20	96.36±1.13	98.50±1.27	0–1.03	0–3.15
	4	96.00±0.05	96.07±0.15	96.06±0.14	0–0.21	0–0.10
	5	99.40±1.26	99.37±1.51	99.37±1.51	0–0.61	0–0.94
	6	97.00±0.82	96.83±0.96	96.83±0.96	0–0.52	0–0.58
	7	96.60±0.52	96.67±0.19	96.67±0.19	0–0.52	0–0.52
	8	99.10±1.29	99.14±1.72	99.20±1.74	0–2.04	0–2.04
	9	98.50±1.35	98.58±1.28	98.58±1.28	0–0.41	0–0.41
	10	99.70±0.95	99.74±0.82	99.80±0.63	0–0.41	0–0.41
Standing	1	98.50±1.35	99.00±1.41	99.00±1.49	0–1.02	0–2.08
	2	97.50±3.06	97.51±3.03	98.17±2.55	0–1.07	0–5.38
	3	100.00±0.10	100.00±0.15	100.00±0.12	0–0.30	0–0.40
	4	97.00±0.05	97.00±0.25	97.00±0.25	0–0.41	0–0.41
	5	99.70±0.95	99.53±1.11	99.53±1.11	0–0.62	0–0.62
	6	96.60±1.07	96.62±1.02	96.62±1.02	0–0.31	0–0.32
	7	97.10±0.32	96.84±0.34	96.84±0.34	0–0.62	0–0.62
	8	99.40±1.26	99.41±1.25	99.41±1.25	0–0.21	0–0.21
	9	99.40±1.26	99.46±1.14	99.46±1.14	0–0.41	0–0.41
	10	99.60±1.26	99.32±2.15	99.32±2.15	0–2.92	0–2.92

**Table 4:** Comparison of the temperatures measured by the commercial device (G-TECH THGTSC3) and the prototype sensor (IR MLX90614), along with the maximum error rates of the prototype relative to the commercial device.

Subject ID	G-TECH THGTSC3 (°C)	IR MLX90614 (°C)	Maximum Error Rate (°C)
1	36.26±0.38	36.18±0.52	0.5
2	36.16±0.05	36.28±0.31	0.7
3	36.00±0.07	35.77±0.21	0.4
4	36.32±0.33	36.31±0.38	0.3
5	36.03±0.12	36.43±0.13	0.5
6	35.96±0.13	36.04±0.21	0.5
7	36.21±0.24	36.30±0.37	0.5
8	36.04±0.07	36.22±0.28	0.5
9	36.03±0.08	36.26±0.22	0.6
10	36.08±0.06	36.42±0.21	0.6

**Table 5:** Blood pressure values for each subject measured by the commercial device GTECH LA800 and estimated by the prototype in the BPT-Finger and BPT-Earlobe configurations, along with the respective error rates relative to the measurements taken with the GTECH LA800 device.

Subject ID	Type	GTECH LA800 (mmHg)	BPT-Finger (mmHg)	BPT-Earlobe (mmHg)	Maximum Error Rate (mmHg)	
					Finger	Earlobe
1	Systolic	125.20±5.53	124.40±4.38	127.50±5.44	2.40	3.40
	Diastolic	78.90±5.20	78.00±4.64	78.60±4.01	2.72	2.52
2	Systolic	124.50±7.17	123.10±4.65	123.80±3.82	3.39	4.10
	Diastolic	79.70±4.14	77.00±5.10	77.80±6.23	3.91	4.24
3	Systolic	136.30±9.10	135.70±8.11	135.80±7.64	1.46	3.38
	Diastolic	79.10±2.60	82.10±5.45	81.40±5.32	1.94	3.26
4	Systolic	123.90±5.24	123.20±4.32	123.40±3.84	1.28	1.27
	Diastolic	71.00±5.40	71.40±3.69	72.50±2.42	1.57	2.89
5	Systolic	117.10±4.56	119.60±1.84	118.90±2.13	3.33	2.62
	Diastolic	81.50±3.89	81.50±3.21	80.30±2.50	2.79	2.57
6	Systolic	115.50±5.64	119.50±6.35	119.90±4.28	4.68	4.48
	Diastolic	68.60±3.86	72.80±2.86	71.40±1.58	4.97	3.28
7	Systolic	163.30±6.67	137.70±4.14	137.70±3.89	25.34	25.34
	Diastolic	71.20±4.13	71.00±3.23	71.40±3.13	0.99	1.00
8	Systolic	106.30±7.94	114.10±5.99	114.10±5.53	8.06	8.08
	Diastolic	76.20±5.05	75.90±4.43	76.50±3.03	4.27	3.36
9	Systolic	136.50±9.94	132.30±7.02	132.50±7.40	4.21	3.81
	Diastolic	82.70±4.35	80.70±4.88	80.80±3.33	3.79	3.33
10	Systolic	105.40±3.03	114.40±4.45	114.40±4.48	9.08	9.08
	Diastolic	79.60±3.69	78.10±2.73	77.90±3.18	1.66	1.87

## Data Availability

The original contributions presented in this study are included in the article. Further inquiries can be directed to the corresponding authors.

## Abbreviations

The following abbreviations are used in this manuscript:

ANVISA	Brazilian Health Regulatory Agency
BLE	Bluetooth Low Energy
BMI	Body Mass Index
BPT	Blood Pressure Trend
BPT-Earlobe	Blood Pressure Trend measured at the Earlobe
BPT-Finger	Blood Pressure Trend measured at the Finger
CBP	Commercial Blood Pressure
CIR	Commercial Infrared Thermometer
COX	Commercial Oximeter
G-TECH LA800	Commercial Blood Pressure Monitor
G-TECH THGTSC3	Commercial Infrared Thermometer
INMETRO	National Institute of Metrology, Quality, and Technology
IoT	Internet of Things
IR	Infrared
IR MLX90614	Infrared MLX90614 Thermometer Sensor
MQTT	Message Queuing Telemetry Transport
PPG	Photoplethysmography
Sp$O_{2}$	Oxygen Saturation
UT-100	Portable Pulse Oximeter (specific commercial device)
